# Expression of CALR mutants causes mpl-dependent thrombocytosis in zebrafish

**DOI:** 10.1038/bcj.2016.83

**Published:** 2016-10-07

**Authors:** K-H Lim, Y-C Chang, Y-H Chiang, H-C Lin, C-Y Chang, C-S Lin, L Huang, W-T Wang, C Gon-Shen Chen, W-C Chou, Y-Y Kuo

**Affiliations:** 1 Graduate Institute of Oncology, National Taiwan University College of Medicine, Taipei, Taiwan; 2 Division of Hematology and Oncology, Department of Internal Medicine, MacKay Memorial Hospital, Taipei, Taiwan; 3 Laboratory of Good Clinical Research Center, Department of Medical Research, MacKay Memorial Hospital, Tamsui District, New Taipei City, Taiwan; 4 Department of Medicine, MacKay Medical College, New Taipei City, Taiwan; 5 Institute of Molecular and Cellular Biology, National Tsing-Hua University, Hsinchu, Taiwan; 6 Division of Hematology, Department of Internal Medicine, National Taiwan University Hospital, College of Medicine, National Taiwan University, Taipei, Taiwan; 7 Department of Laboratory Medicine, National Taiwan University Hospital, Taipei, Taiwan

## Abstract

*CALR* mutations are identified in about 30% of *JAK2*/*MPL*-unmutated myeloproliferative neoplasms (MPNs) including essential thrombocythemia (ET) and primary myelofibrosis. Although the molecular pathogenesis of *CALR* mutations leading to MPNs has been studied using *in vitro* cell lines models, how mutant CALR may affect developmental hematopoiesis remains unknown. Here we took advantage of the zebrafish model to examine the effects of mutant CALR on early hematopoiesis and model human *CALR*-mutated MPNs. We identified three zebrafish genes orthologous to human *CALR*, referred to as *calr*, *calr3a* and *calr3b*. The expression of CALR-del52 and CALR-ins5 mutants caused an increase in the hematopoietic stem/progenitor cells followed by thrombocytosis without affecting normal angiogenesis. The expression of CALR mutants also perturbed early developmental hematopoiesis in zebrafish. Importantly, morpholino knockdown of *mpl* but not *epor* or *csf3r* could significantly attenuate the effects of mutant CALR. Furthermore, the expression of mutant CALR caused jak-stat signaling activation in zebrafish that could be blocked by JAK inhibitors (ruxolitinib and fedratinib). These findings showed that mutant CALR activates jak-stat signaling through an mpl-dependent mechanism to mediate pathogenic thrombopoiesis in zebrafish, and illustrated that the signaling machinery related to mutant CALR tumorigenesis are conserved between human and zebrafish.

## Introduction

The *BCR-ABL*-negative classic myeloproliferative neoplasms (MPNs) are clonal hematopoietic stem cell disorders including polycythemia vera, essential thrombocythemia (ET) and primary myelofibrosis (PMF).^1^ The *JAK2*V617F and *MPL* exon 10 mutations are two important driver mutations in MPNs and cause the activation of the JAK-signal transducer and activator of transcription (STAT) signaling that is central to the pathogenesis of MPNs.^2^ Calreticulin (CALR) is a 46-kDa highly conserved, multicompartmental and multifunctional protein.^3^ CALR has its role as a Ca^2+^-binding chaperone protein and acts in concert with calnexin to ensure proper protein and glycoprotein folding in the endoplasmic reticulum (ER).^4^ Recently, two research groups discovered *CALR* mutations in about 30% of *JAK2* and *MPL-*unmutated ET and PMF patients.^5, 6^ All *CALR* mutations are indels mutations in exon 9 and cause +1 base frameshift generating a novel C-terminus characterized by the loss of the ER retention signal KDEL and the change from acidic to basic amino-acid sequence. Although there are >50 *CALR* mutants identified in MPNs, the most prevalent types of *CALR* mutations are a 52 bp deletion (L367fs*46, type 1 mutation, CALR-del52) and a 5 bp insertion of TTGTC (K385fs*47, type 2 mutation, CALR-ins5) accounting for >80% of all patients with mutant *CALR*.^5, 6^ Most *CALR* mutations are mutually exclusive with the *JAK2* and *MPL* mutations, but some patients were found to have *JAK2* and *CALR* co-mutations.^7^ ET and PMF patients with *CALR* mutations have been found to have different clinical characteristics such as younger age and higher platelet count and to carry a better prognosis than those patients with *JAK2*V617F mutation.^7, 8, 9, 10^

Recent studies have focused on the underlying mechanism of *CALR* mutations in the pathophysiology of MPNs. With the use of *in vitro* cell lines and retroviral mouse models, *CALR* mutants were found to activate the JAK-STAT signaling in an MPL-dependent manner.^11, 12, 13, 14, 15^ Although the expression of *CALR* mutants resulted in pathogenic thrombocytosis in adult mice, whether *CALR* mutants may disrupt normal hematopoiesis during early development remains unknown. The zebrafish is a useful disease model system and has been successfully utilized in studying hematopoiesis and leukemogenesis.^16, 17, 18, 19, 20^ The early hematopoietic system in zebrafish involves two distinct primitive and definitive waves of development that is rapidly established within a few days after fertilization.^18^ The developmental hematopoiesis of zebrafish also shows broad conservation with mammalian species and is regulated by conserved molecular pathways.^18^ The transparency of zebrafish at the embryonic and larval stages has made it suitable for direct observation of the hematopoietic process. In addition, zebrafish can be used for *in vivo* high throughput screening due to its good permeability to chemical added to water during early developmental stages.^21, 22, 23^ Here we aimed to evaluate the pathophysiologic effects of mutant CALR during embryonic hematopoietic development and to test the therapeutic effects of JAK inhibitors on mutant CALR using the *in vivo* zebrafish model.

## Materials and methods

### Zebrafish husbandry

Wild-type AB strain of zebrafish (*Danio rerio*) and the transgenic lines Tg(*cd41*:GFP)^24^ and Tg(*fli1*:EGFP)^25^ were maintained and manipulated with standard measure as previously described.^26^ The stages of embryonic development were determined based on Kimmel *et al.*^27^ Pigmentation was blocked by using 0.003% 1-phenyl-2-thiourea in some experiments. For pharmacologic inhibition, embryos were incubated with ruxolitinib (Abmole Bioscience, Houston, TX, USA) or fedratinib (Abmole Bioscience) from 1–2 cells stage to 5 days post fertilization (d.p.f.) with or without microinjection of *CALR* mRNA. The zebrafish experiments were approved by the MacKay Memorial Hospital Animal Care and Use Committee.

### Identification of zebrafish ortholog of human CALR

Human genes located in 19p13.11-13.2 were identified using the National Center for Biotechnology Information (NCBI) Map Viewer.^28^ Genes surrounding the three zebrafish *calr* genomic regions were identified using Ensembl^29^ and Synteny database.^30^ Human CALR protein sequence was used to BLASTP against zebrafish GRCz10 using the Ensembl platform (Ensembl release 82).^29^ Alignment and comparative analysis between protein sequences was performed using the Clustal Omega algorithm^31^ and edited by GeneDoc.^32^

### Human and zebrafish CALR cDNAs cloning and mRNA synthesis

Full-length CALR cDNAs were subcloned in the pCS2^+^ vector and into a bicistronic pSYC-102 T2A vector (a gift from Dr Seok-Yong Choi) replacing the mCherry-CAAX reporter gene using the In-Fusion Cloning Kit (Clontech, Mountain View, CA, USA) (Supplementary Figure S1).^33^ All vector sequences were verified by sequencing. The mMessage mMachine SP6 kit (Ambion, Austin, TX, USA) was used for *in vitro* transcription of capped mRNAs from vectors according to the manufacturer's protocol. mRNAs from the bicistronic pSYC-102-CALR vectors were only used to express EGFP and CALR concurrently in wild-type zebrafish embryos and only embryos expressing green fluorescence were collected under fluorescence microscope for use in the reverse transcription and real-time PCR.

### Morpholinos and microinjection

Morpholinos (MOs) blocking splicing of *mpl* and *epor*, and translation (ATG/5′UTR) of *csf3r* were purchased from Gene Tools (Philomath, OR, USA; MO sequences are listed in Table 1).^24, 34, 35^ Standard control MO was used as negative control. Embryos at the 1–2 cells stage were injected with MO (1 ng) or mRNAs (100 pg). Co-injection of each MO and *CALR* mutant mRNA was performed in a subset of embryos.

### Reverse transcription and real-time PCR

Total RNA was extracted from embryos using miTotal Miniprep System (Viogene, New Taipei City, Taiwan) and reverse transcribed using a High Capacity cDNA Reverse Transcription Kits (Applied Biosystems, Foster City, CA, USA). Primer sequences are listed in Supplementary Table 1. Fast SYBR Green Master Mix (Applied Biosystems) was used for real-time quantitative PCR according to the manufacturer's instructions.

### Western blotting

Total proteins were extracted from zebrafish embryos at 24 h post fertilization (h.p.f.). Equal amounts of protein were denatured and electrophoresed. Membranes were immunoblotted with the following primary antibodies: CALR (Abcam, Cambridge, UK; recognizing N-terminal sequences of both human and zebrafish wild-type CALR proteins), gapdh and customized mutant CALR (GeneTex, Hsinchu City, Taiwan; specifically recognizing human CALR exon 9 indel mutant protein sequence), STAT5 (Santa Cruz, Dallas, TX, USA) and phospho-STAT5 (Cell Signaling, MA, USA).

### Imaging

Live embryos were imaged using a Leica MSV269 fluorescence stereomicroscope and photographed using a Leica DFC425 C digital camera and Leica Application Suite software (Leica Microsystems, Wetzlar, Germany). GraphPad Prism 7 software and ImageJ (National Institutes of Health) were used to process images.

### Statistical analysis

The Student *t-*test or analysis of variance test were used. Data were expressed as mean±s.e.m. Significance was determined at *P<0.05, **P<0.01 and ***P<0.001.

## Results

### Zebrafish ortholog of human CALR

**Figure 1:**
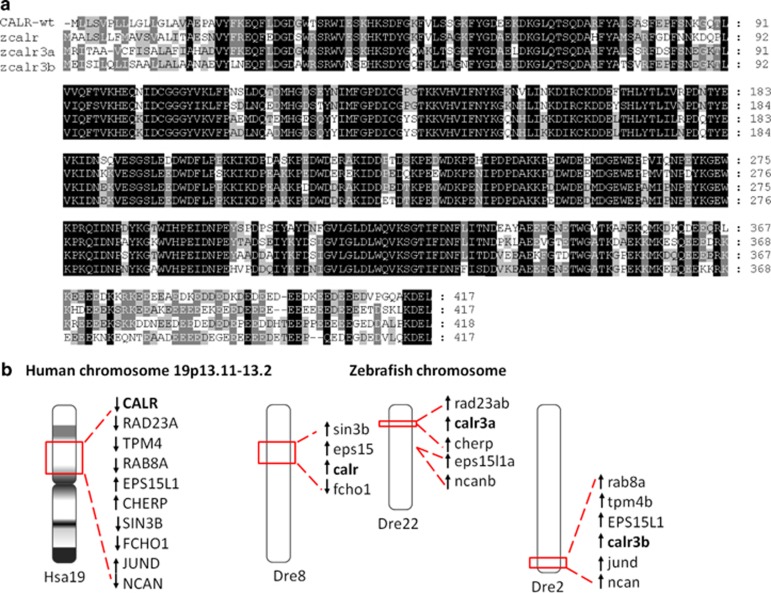
Identification of three zebrafish *calr* genes. (**a**) Alignment of human CALR (top row), zebrafish calr (second row), calr3a (third row) and calr3b (bottom row) protein sequences. The regions of sequence identity in the four proteins are shaded. (**b**) The genomic loci surrounding human *CALR* on chromosome 19p13.2 (Hsa19) are syntenic with the regions where zebrafish *calr* (on chromosome 8, Dre8), *calr3a* (on chromosome 22, Dre22) and *calr3b* (on chromosome 2, Dre2) are located in the zebrafish genome, respectively.

To search for the zebrafish ortholog of human *CALR* gene, human CALR protein sequence was used to BLASTP against zebrafish GRCz10 (Ensembl release 82). We identified three annotated zebrafish orthologs of the human *CALR* gene (ENSG000001792), *calr* (ENSDARG00000076290 at chromosome 8), *calr3a* (ENSDARG00000103979 at chromosome 22) and *calr3b* (ENSDARG00000102808 at chromosome 2). After comparative analysis using the Clustal Omega algorithm, the amino-acid sequence of zebrafish calr, calr3a and calr3b proteins shares an overall 75%, 71% and 70% identity to human CALR protein sequence, respectively. The three functional domains in CALR are conserved in all three zebrafish calr proteins, including the KDEL ER retention signal at the C-terminus (Figure 1a). In addition, the genomic loci surrounding human chromosome 19p13.2 containing the *CALR* gene are syntenic with the regions of zebrafish *calr* on chromosome 8, *calr3a* on chromosome 22 and *calr3b* on chromosome 2 based on the search in NCBI Map Viewer, Ensembl database and Synteny database (Figure 1b). These results indicated that the three zebrafish *calr* genes are likely true orthologs of human *CALR*.

### Effects of mutant CALR expression on thrombopoiesis and angiogenesis in zebrafish

**Figure 2:**
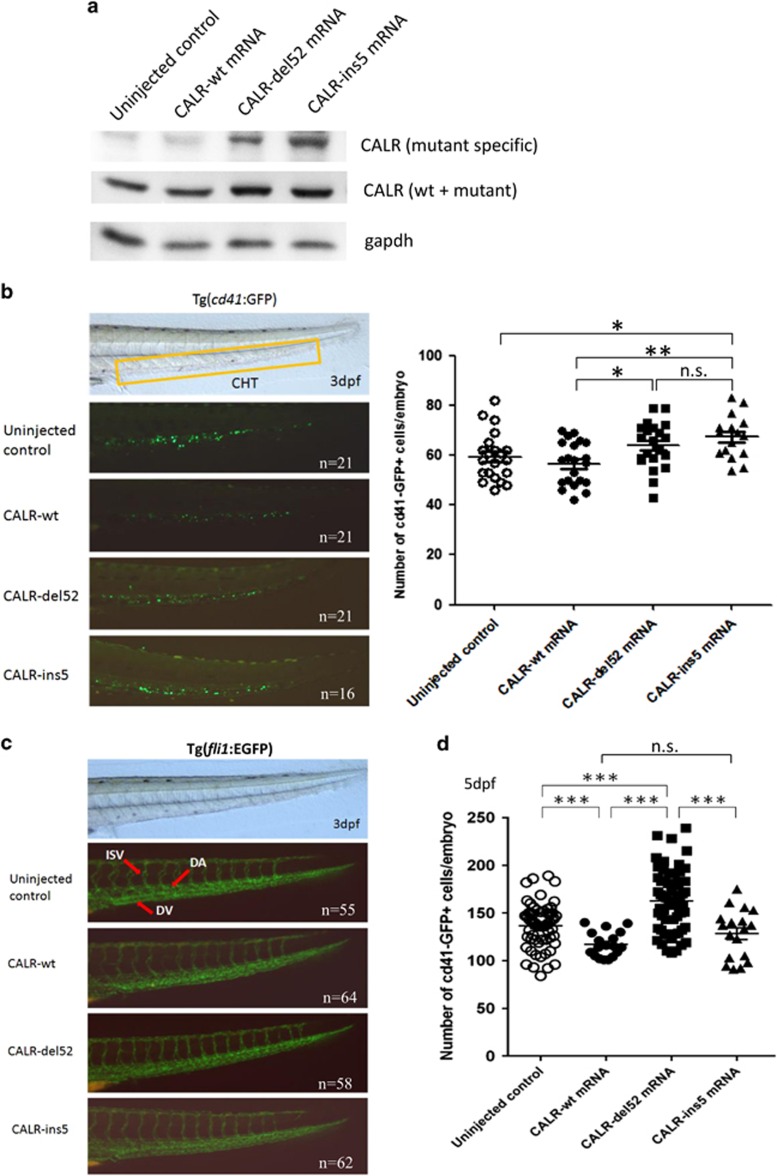
Effects of the expression of mutant CALR on the number of hematopoietic stem/progenitor cells and angiogenesis. (**a**) CALR protein expression in uninjected, *CALR*-wt-, *CALR*-del52- or *CALR*-ins5-injected embryos at 24 h.p.f. gapdh was used to normalize the total amount of protein in each sample. The expression of human CALR mutant proteins was confirmed in *CALR*-del52- and *CALR*-ins5-injected embryos by a customized human CALR mutant-specific polyclonal antibody (top panel). (**b**) Brightfield image (left panel) of a 3 d.p.f. embryo with a box area indicating caudal hematopoietic tissue where CD41^+^ cells were counted. Green cells in the darkfield images (left panel) indicated expression of GFP under the control of the *cd41* promoter, and were counted and showed on the right panel. (**c**) The development of dorsal aorta (DA), dorsal vein (DV) and intersegmental vessel (ISV) as indicated by the red arrows (top panel) in uninjected, *CALR*-wt-, *CALR*-del52- or *CALR*-ins5-injected embryos of the Tg(*fli1*:EGFP) line at 3 d.p.f. (**d**) The total numbers of CD41^+^ thrombocytes were counted in uninjected, *CALR*-wt-, *CALR*-del52- or *CALR*-ins5-injected embryos of the Tg(*cd41*:GFP) line at 5 d.p.f. (the direction of embryos was anterior to the left, dorsal upwards, lateral view; n.s., not significant; *P<0.05, **P<0.01, ***P<0.001; Student *t-*test). The number of embryos used in each experiment is indicated by ‘n' in figures.

For the expression of mRNA in zebrafish embryo, we first performed a dose-finding study ranging from 50 to 200 pg *CALR* mRNA. Phenotype could be observed at dose of 100 pg mRNA per embryo which was compatible with normal development for most embryos. All CALR proteins were adequately expressed at comparable amount at dose of 100 pg (Figure 2a, middle panel). The expression of CALR-del52 and CALR-ins5 mutant proteins was also confirmed by mutant CALR-specific antibody (Figure 2a, top panel). Therefore, 100 pg mRNA was injected throughout the study. To determine whether mutant CALR had an effect on hematopoietic stem and progenitor cells (HSPCs) in zebrafish, we injected the three mRNAs encoding *CALR* wild-type (CALR-wt), *CALR*-del52, and *CALR*-ins5 into 1–2 cells stage embryos of the *cd41*:GFP line, and the numbers of CD41^+^ cells in the caudal hematopoietic tissue (CHT) at 3 d.p.f. indicating the HSPCs were counted.^24^ Expression of both *CALR*-del52 and *CALR*-ins5 mutant mRNA significantly increased the numbers of HSPCs in the CHT when compared with *CALR*-wt mRNA (Figure 2b). However, the numbers of HSPCs did not have statistically significant difference between *CALR*-del52 and *CALR*-ins5 mutant groups at this developmental stage. To ascertain that the increase of HSPCs was not affected by the change in angiogenesis during early development, mRNAs encoding *CALR*-wt, *CALR*-del52, and *CALR*-ins5 were injected into 1–2 cells stage embryos of the *fli1*:EGFP line. No obvious changes in the angiogenesis were visualized in CALR-wt and mutant CALR expressing embryos at 3 d.p.f. when compared with uninjected control (Figure 2c). To determine whether CALR had an effect on mature thrombocyte, the numbers of CD41^+^ thrombocytes in the *cd41*:GFP line were counted at 5 d.p.f. after injection. Mutant *CALR*-del52 significantly increased the number of CD41^+^ thrombocytes (mean 162.5±4.1 per embryo) when compared with *CALR*-wt (mean 117.1±3.1 per embryo, *P*<0.001), mutant *CALR*-ins5 (mean 128.3±6.1 per embryo, *P*<0.001) and uninjected control (mean 136.7±3.0 per embryo, *P*<0.001; Figure 2d). Although mutant *CALR*-ins5 slightly increased the number of CD41^+^ thrombocytes when compared with *CALR*-wt, there was no statistically significant difference. Altogether, our data demonstrated that the effect of mutant CALR on thrombopoiesis in zebrafish is dependent on the presence of the novel C-terminus and is also related to specific CALR mutant protein sequences.

### Mutant CALR requires mpl to cause thrombocytosis in zebrafish

**Figure 3:**
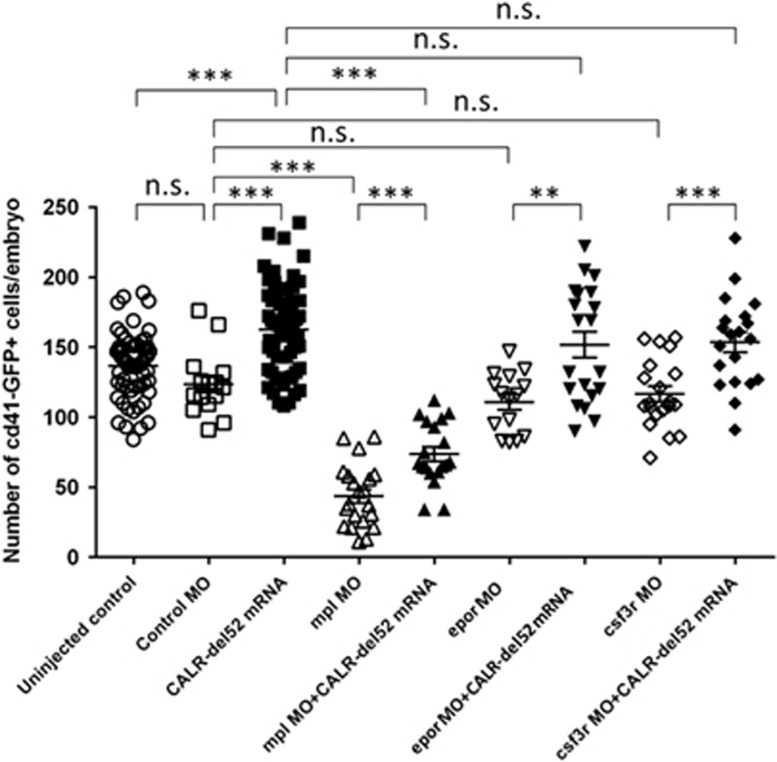
Mutant CALR requires mpl to cause thrombocytosis in zebrafish. Morpholinos (MOs) targeting *mpl*, *epor* or *csf3r* (1 ng per embryo) were injected into 1–2 cells stage embryos of the Tg(*cd41*:GFP) line with or without co-injection of *CLAR*-del52 mRNA (100 pg). Standard control MO was used as negative control. The total numbers of CD41^+^ thrombocytes were counted at 5 d.p.f. and compared as indicated. The number of CD41^+^ thrombocytes significantly decreases upon *mpl* knockdown when compared with the control MO group as well as the *CALR*-del52 group. Co-injection of *CALR*-del52 mRNA can only partially reverse the knockdown effect of *mpl* MO. When *CALR*-del52 mRNA was co-injected with *epor* MO or *csf3r* MO, the numbers of CD41^+^ thrombocytes were comparable to those of *CALR*-del52-injected embryos (n.s., not significant; **P<0.01, ***P<0.001; Student *t-*test).

To test whether cytokine receptors are involved in the pathogenesis of thrombocytosis caused by mutant CALR in zebrafish, *mpl*, *epor* and *csf3r* MOs (each with 1 ng) were injected in 1–2 cells stage embryos of *cd41*:GFP line and assayed for their effects on the number of CD41^+^ thrombocytes at 5 d.p.f. Co-injection of *CALR*-del52 mutant mRNA (100 pg) with each MO was also performed in a subset of embryos. At 5 d.p.f., the number of CD41^+^ thrombocytes significantly decreased upon *mpl* knockdown (mean 43.6±4.9 per embryo) when compared with the control MO group (mean 123.5±5.9 per embryo, *P*<0.001) and the mutant *CALR*-del52 group (*P*<0.001; Figure 3). Importantly, co-injection of *CALR*-del52 mutant mRNA (mean 73.7±5.1 per embryo) can only partially reverse the knockdown effect of *mpl* MO. In contrast, the numbers of CD41^+^ thrombocytes did not decrease significantly upon *epor* MO (mean 110.6±5.5 per embryo) or *csf3r* MO (mean 116.6±5.6 per embryo) knocked down compared with the control MO group. When *CALR*-del52 mutant mRNA was co-injected with *epor* (mean 151.7±9.2 per embryo) or *csf3r* (mean 153.6±7.2 per embryo) MOs, the numbers of CD41^+^ thrombocytes were comparable to those of *CALR*-del52–injected embryos (both *P*=0.3). Collectively, these findings indicated that the expression of mutant CALR causes thrombocytosis through an mpl-dependent mechanism in zebrafish.

### Effects of CALR mutants on lineage-specific and cytokine gene expression

The increase in thrombopoiesis upon expression of mutant CALR prompted us to evaluate their effects on hematopoietic lineage-specific, thrombopoiesis,^36^ cytokine and cytokine receptor gene expression in zebrafish embryos at 3 d.p.f. The expression of HSC gene *runx1* was significantly upregulated in *CALR*-ins5 group but was modestly downregulated in *CALR*-del52 group (Table 2). Also, the expression of *c-myb* and *scl* was only downregulated in *CALR*-del52 group. Although *gata1* was modestly downregulated in mutant CALR groups, the expression of *α-eHb* and *β-eHb* was not affected by both CALR mutants. The expression of early (*spi1b)* and late myeloid (*mpo*: granulocytic; *l-plastin*: macrophage) lineage genes, *epo* and *epor* showed no significant changes. However, the expression of lymphoid lineage genes (*rag1*, *rag2* and *lck*) was modestly downregulated in mutant CALR groups. Although the expression of *mpl* was significantly downregulated in both mutant CALR groups, both *tpo* and *csf3r* expressions were only downregulated in *CALR*-del52 group. In the group of genes related to thrombopoiesis, only the expression of *nbeal2* was significantly downregulated in *CALR*-del52 group.

### Effects of mutant CALR on jak-stat signaling in zebrafish

**Figure 4:**
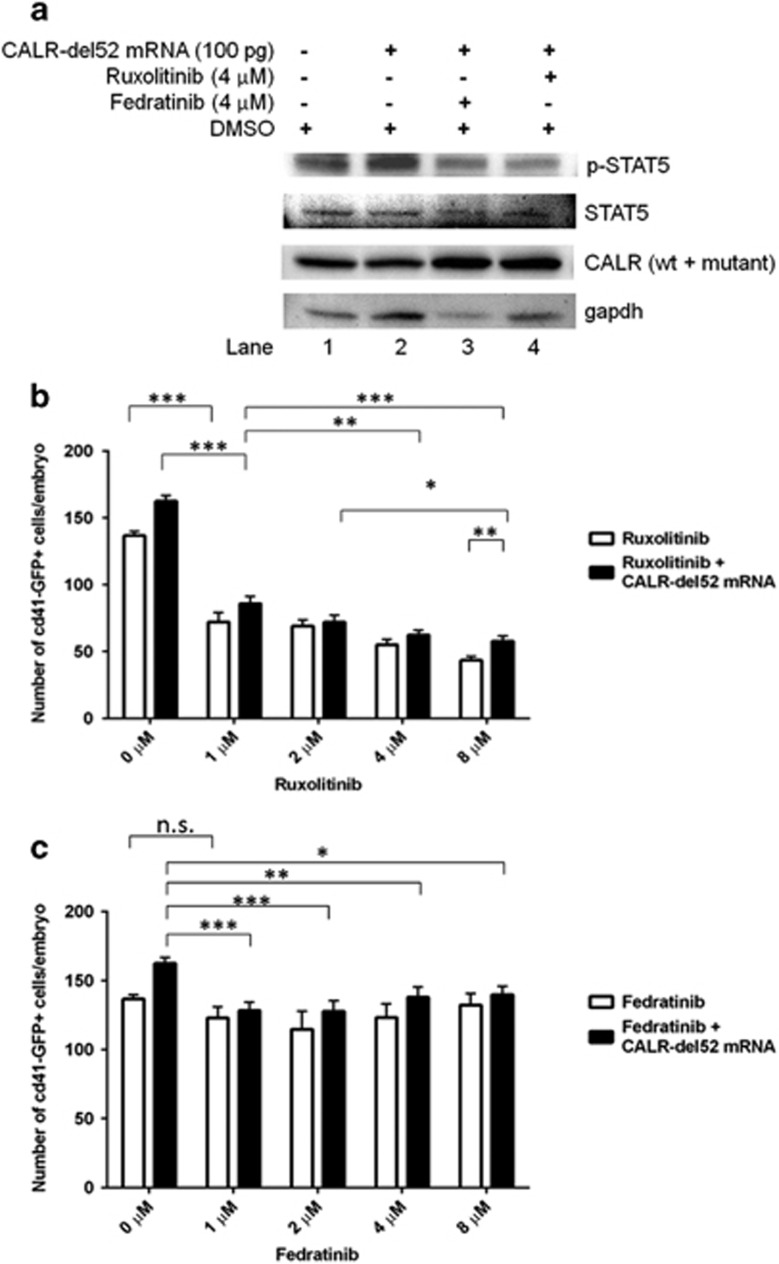
The expression of mutant CALR activates jak-stat signaling in zebrafish. (**a**) Western blotting showing embryos of wild-type zebrafish injected with *CALR*-del52 mRNA (100 pg) have significantly increased signal transducer and activation of transcription (stat) 5 phosphorylation (lane 2) as compared with uninjected control embryos from the same batch at 24 h.p.f. (lane 1). Pharmacologic treatment of embryos with a JAK2-selective inhibitor (fedratinib) and a dual JAK1/JAK2 inhibitor (ruxolitinib) significantly attenuated the enhanced stat5 phosphorylation induced by *CALR*-del52 mRNA stat5 phosphorylation (lane 3 and 4, respectively). Total amount of stat5 protein was not affected in all experiments. (**b** and **c**) Effects of the pharmacologic treatment with ruxolitinib and fedratinib (from 1 μM to 8 μM) on the numbers of CD41^+^ thrombocytes at 5 d.p.f. with or without the injection of *CALR*-del52 mRNA. Treatment with ruxolitinib significantly decreases the numbers of CD41^+^ thrombocytes in uninjected control as well as *CALR*-del52-injected embryos in a dose-dependent manner. Whereas treatment with fedratinib has minimal and insignificant inhibitory effect on the number of CD41^+^ thrombocyte in uninjected control embryos, and has a modest and significant dose-independent inhibitory effect on mutant CALR-induced thrombocytosis (n.s., not significant; *P<0.05, **P<0.01, ***P<0.001; Student *t*-test).

We then investigated whether the expression of mutant CALR can activate the jak-stat signaling in zebrafish. The expression of *CALR*-del52 mRNA significantly increased stat5 phosphorylation (Figure 4a, lane 2). Furthermore, treatment with ruxolitinib and fedratinib significantly ameliorated the enhanced stat5 phosphorylation induced by *CALR*-del52 mRNA (Figure 4a, lane 3 and 4). In addition, treatment with ruxolitinib significantly decreased the numbers of CD41^+^ thrombocytes in uninjected control as well as *CALR*-del52-injected embryos in a dose-dependent manner (Figure 4b). Whereas treatment with fedratinib only had minimal inhibitory effect on the number of CD41^+^ thrombocytes in uninjected control embryos, and had a modest and significant dose-independent inhibitory effect on mutant CALR-induced thrombocytosis (Figure 4c). Our results demonstrated that mutant CALR-mediated pathogenic thrombopoiesis involves jak-stat activation that can be blocked by JAK inhibitors.

## Discussion

In this study, we have used the zebrafish animal model to examine the pathogenesis of mutant CALR in MPNs. We first identified three zebrafish orthologs for human *CALR* gene. We have shown that expression of the CALR-del52 mutant disturbs thrombopoiesis and increases the number of HSPCs in the CHT followed by significant thrombocytosis in the zebrafish embryo. These findings are consistent with the myeloproliferative phenotype in retroviral mouse bone marrow transplantation models elicited by mutant CALR expression characterized by thrombocytosis and megakaryocytic hyperplasia recapitulating those seen in patients with ET and myelofibrosis.^12, 14^

The highly conserve protein sequences between human *CALR* and the three zebrafish *calr* genes suggested functional conservation between human and zebrafish *CALR*. Ma *et al.*^37^ recently reported that MO knockdown of *calr* perturbs myeloid and HSCs lineages during zebrafish embryonic development including a decrease in the expression of genes associated with myeloid lineages at 24 h.p.f. and an increase in the expression of *cmyb* at 48, 72 and 96 h.p.f. We have also shown that the expression of genes involved in lineage-specific hematopoiesis, thrombopoiesis, cytokines and cytokine receptors could be perturbed by the expression of mutant CALR in zebrafish during early development. These data suggested that zebrafish *calr* genes have an important role in the regulation of vertebrae hematopoiesis. In addition, our data suggested that mutant CALR does not promote thrombopoiesis through the upregulation of *mpl* and *tpo* levels. Rather, the downregulation of *mpl* and *tpo* might represent a negative-feedback mechanism related to increased thrombopoiesis due to mutant CALR expression.

On the basis of the data from the murine and zebrafish animal models, the causative relationship between CALR mutations and thrombocytosis can be confirmed, and *CALR* mutations have been established as one of the driver mutations in MPNs. Furthermore, we demonstrated that expression of *CALR*-del52 (type 1 mutation) causes higher thrombocyte count than *CALR*-ins5 (type 2 mutation) at 5 d.p.f. in zebrafish embryo. Similar finding has been reported in murine model that marked thrombocytosis was rapidly induced in *CALR*-del52-expressing mice and then progressed to myelofibrosis, and *CALR*-ins5-expressing mice only developed modest thrombocytosis resembling mild ET phenotype.^12^ This is consistent with the clinical finding that *CALR*-del52 mutation is more frequently detected in PMF than in ET,^5^ and also confirms the differential effects of *CALR* variants on thrombopoiesis and clinical phenotypes.^10, 38, 39^

To further elucidate the molecular pathogenesis of mutant *CALR* in our zebrafish model, we have used MO knockdown experiments to show that only the *mpl* MO can significantly attenuate the effect of mutant CALR on thrombopoiesis. Both *epor* and *csf3r* MOs were not able to inhibit the effect of mutant CALR. These findings indicated that mpl has an essential and specific role required by mutant CALR to cause thrombocytosis in zebrafish. Because CALR is physiologically functioning as a chaperone for MPL, it is reasonable to speculate that mutant CALR may interact directly with mpl to cause thrombocytosis in zebrafish. Our data are consistent with those recently reported by several groups of researchers using *in vitro* cell line models.^11, 12, 13, 14, 15^ In these studies, both the novel C-terminus of CALR mutants and the direct interaction of mutant CALR with MPL receptor are required to activate MPL and the downstream JAK-STAT signaling, which in turn is responsible for cytokine-independent growth of Ba/F3-MPL and UT-7/TPO cell lines. Chachoua *et al.*^13^ reported that the specific activation of MPL receptor by mutant CALR required both the presence of extracellular *N*-glycosylation residues of MPL and the glycan-binding site at the novel C-terminus of mutant CALR. In addition, Elf *et al.*^14^ reported that the physical interaction between mutant CALR and MPL is dependent on the positive electrostatic charge of the C-terminus of the mutant CALR but not dependent on specific novel C-terminal sequence. Recently, Balligand *et al.*^40^ reported similar finding that highly similar but not identical murine *Calr* exon 9 frameshift mutants also require Mpl interaction to activate the JAK-STAT signaling. Moreover, the positive charge predominant novel C-terminus of the mutant CALR results in different calcium-binding capacity, which may alter calcium homeostasis and signaling processes in mutant cells. All these structural differences and changes will likely contribute to the different clinical phenotypes seen in different *CALR* variants.^10, 38, 39, 41^

We have also demonstrated that the expression of human CALR mutant is able to activate jak-stat signaling in zebrafish. In addition, jak-stat signaling in zebrafish can also be inhibited by JAK2 inhibitors used in clinical trials illustrating that the conserved signaling machinery in human and zebrafish.^42, 43, 44^ Our data showed that ruxolitinib treatment results in a dose-dependent inhibitory effect on both normal thrombopoiesis and thrombocytosis caused by mutant CALR in zebrafish. By contrast, JAK2-selective inhibitor fedratinib has only minimal inhibitory effects on normal thrombopoiesis but has modest and dose-independent inhibitory effect on thrombocytosis caused by mutant CALR. Our data suggested that fedratinib can normalize the thrombocytosis caused by the expression of mutant CALR and does not cause significant thrombocytopenia in zebrafish model. These observations are comparable to the findings that both ruxolitinib and fedratinib have been demonstrated to have clinical responses in MPN patients harboring *CALR* mutations.^45, 46, 47^ However, fedratinib has less hematological toxicities than ruxolitinib especially thrombocytopenia, which is a dose-limiting toxicity of ruxolitinib.^42, 43, 44^ Despite both JAK inhibitors are effective in the reduction of splenomegaly and the relief of clinical symptoms, they are not likely to substantially modify the natural history of the *BCR*-*ABL*-negative classic MPNs including *CALR*-mutated PMF. Importantly, these JAK inhibitors are not specifically designed for *JAK2*V617F mutation. However, the unique pathogenic mechanism of mutant CALR in MPNs has led to the possibility of new therapeutic approach targeting the interaction and binding between mutant CALR and MPL. In this regard, our results highlight the advantage and support the use of zebrafish as a relevant *in vivo* whole-organism model for the testing and screening of therapeutic compounds targeting mutant CALR.^22^

In conclusion, we have used the zebrafish model to show that mutant CALR promotes the activation of jak-stat signaling through an mpl-dependent mechanism to mediate pathogenic thrombopoiesis during zebrafish early hematopoiesis. These findings are consistent with those observed in *in vitro* cell line and mouse models and illustrated that the signaling machinery related to mutant CALR tumorigenesis are conserved between human and zebrafish. Zebrafish has also been shown to be a relevant *in vivo* model for the development of novel therapeutic compounds targeting mutant CALR. Future studies using stable mutant CALR transgenic or knock-in zebrafish models for this purpose will be warranted.

## Figures and Tables

**Table 1:** Morpholino sequences for *mpl*, *epor* and *csf3r* knockdown

*Gene*	*Target*	*ZFIN identity*	*MO sequence*
*mpl*	Intron1/exon2 boundary of exon 2	ZDB-MRPHLNO-060421-1	5′-CAGAACTCTCACCCTTCAATTATAT-3′
*epor*	Intron1/exon2 boundary of exon 2	ZDB-MRPHLNO-080325-2	5′-AACTGGGCCACTGAACAATCAAATT-3′
*csf3r*	ATG/5′UTR	ZDB-MRPHLNO-111213-1	5′-GAAGCACAAGCGAGACGGATGCCAT-3′
Standard control	Human beta-globin intron mutation	NA	5′-CCTCTTACCTCAGTTACAATTTATA-3′

Abbreviations: NA, not applicable; ZFIN, Zebrafish International Resource Center.

**Table 2:** Effects of *CALR* mutant mRNA injection on the expression of genes in zebrafish embryo genes involved in lineage-specific hematopoiesis, thrombopoiesis, cytokines and cytokine receptors were examined based on real-time quantitative PCR of zebrafish embryos at 3 days post fertilization, with reference to that of *CALR* wild-type mRNA injection

*Category*	*Gene*	*CALR-wt*^a^	*CALR-del52 Mean±s.e.m.*	*CALR-ins5 Mean±s.e.m.*	*CALR-wt* vs *CALR-del52* vs *CALR-ins5* P-*value*^b^	*CALR-wt* vs *CALR-del52* P*-value*^c^	*CALR-wt* vs *CALR-ins5* P*-value*^c^	*CALR-del52* vs *CALR-ins5* P-*value*^c^
Hematopoietic stem cells	*cmyb*	1.00	0.73±0.04	1.07±0.02	<0.001	0.02	NS	0.001
	*runx1*	1.00	0.78±0.06	1.20±0.06	0.003	NS	0.029	0.008
Hemangioblast	*scl*	1.00	0.75±0.08	0.91±0.05	0.043	NS	NS	NS
	*lmo2*	1.00	0.85±0.06	0.99±0.09	NS	NS	NS	NS
Erythropoiesis	*gata1*	1.00	0.87±0.01	0.89±0.02	0.002	0.012	0.036	NS
	*α eHb*	1.00	0.82±0.11	0.89±0.11	NS	NS	NS	NS
	*β eHb*	1.00	0.73±0.12	0.96±0.22	NS	NS	NS	NS
Vasculature	*fli1*	1.00	0.76±0.06	0.84±0.08	NS	0.017	NS	NS
Early myelomonocytic lineage	*spi1b*	1.00	1.13±0.10	1.01±0.10	NS	NS	NS	NS
Late myelomonocytic lineage	*L-plastin*	1.00	0.99±0.17	0.94±0.09	NS	NS	NS	NS
Lymphoid lineage	*rag1*	1.00	0.56±0.07	1.01±0.12	0.012	0.003	NS	0.033
	*rag2*	1.00	0.96±0.07	0.84±0.03	NS	NS	0.01	NS
	*lck*	1.00	0.50±0.06	1.08±0.13	0.006	0.015	NS	0.017
Thrombopoiesis	*arhgef3*	1.00	0.80±0.09	0.90±0.07	NS	NS	NS	NS
	*emilin1a*	1.00	0.90±0.03	0.91±0.04	NS	0.036	NS	NS
	*nbeal2*	1.00	0.64±0.10	1.08±0.06	0.007	NS	NS	0.019
	*max*	1.00	0.88±0.03	0.87±0.06	NS	0.021	NS	NS
Cytokines and cytokine receptors	*tpo*	1.00	0.88±0.05	1.12±0.05	0.016	NS	NS	0.026
	*mpl*	1.00	0.57±0.05	0.52±0.06	0.001	0.014	0.002	NS
	*epo*	1.00	0.99±0.10	1.12±0.23	NS	NS	NS	NS
	*epor*	1.00	0.80±0.10	0.96±0.09	NS	NS	NS	NS
	*mpo*	1.00	0.85±0.09	0.84±0.06	NS	NS	NS	NS
	*csf3r*	1.00	0.62±0.04	1.15±0.14	0.01	0.001	NS	0.021

Abbreviations: ANOVA, analysis of variance; NS, not significant; wt, wild-type.

Data are from triplicate results.

a Data of *CALR*-wt was arbitrarily set to 1.00 in all cases.

b ANOVA test.

c Student *t*-test.
