# Supplementary material for: Expression of CALR mutants causes mpl-dependent thrombocytosis in zebrafish
Source: Blood Cancer J. 2016 Oct 7;6(10):e481–. doi: 10.1038/bcj.2016.83 (PMC5098260; doi:10.1038/bcj.2016.83)
Supplement: Supplementary Information [file bcj201683x1.doc]

**Supplementary materials:**

**A. Supplementary Methods**

Human and zebrafish CALR cDNAs

Full-length wild-type *CALR* cDNA was cloned from K562 cells into T&A™ Cloning Vector (Yeastern Biotech Co., Taipei, Taiwan) according to the manufacturer’s protocol (Forward primer: 5'-GATC**CTCGAG**ATGCTGCTATCCGTGCCGCTGC-3'; reverse primer: 5'-GATC**GAATTC**CTACAGCTCGTCCTTGGCCTGGC-3'; restriction enzyme sites in bold letters: *Xho*I-*Eco*RI). Human *CALR* type 1 (CALR-del52) and type 2 (CALR-ins5) mutated cDNAs were obtained by custom gene synthesis with a His-tag in the N-terminal.

**B. Supplementary Table** S1. Real-time quantitative polymerase chain reaction primer sequences

| ZF β-actin_qF | 5'-ATTGCTGACAGGATGCAGAAG |
| --- | --- |
| ZF β-actin_qR | 5'-GATGGTCCAGACTCATCGTACTC |
| ZF scl_qF | 5'-CTATTAACCGTGGTTTTGCTGG |
| ZF scl_qR | 5'-CCATCGTTGATTTCAACCTCAT |
| ZF lmo2_qF | 5'-GGACGCAGGCTTTACTACAAAC |
| ZF lmo2_qR | 5'-CCGGATCCTCTTTTCACAGGAA |
| ZF CD41_qF | 5'-CTGAAGGCAGTAACGTCAAC |
| ZF CD41_qR | 5'-TCCTTCTTCTGACCACACAC |
| ZF cmyb_qF | 5'-AGGGAATCGTCTGCTCTTCCG |
| ZF cmyb_qR | 5'-CAGCAGTTGAACACATGGGAAC |
| ZF runx1_qF | 5'-CCGACAGAAGCCGGATGA |
| ZF runx1_qR | 5'-TGGCACTTCGCCTCAACTG |
| ZF gata1_qF | 5'-AAGATGGGACAGGCCACTAC |
| ZF gata1_qR | 5'-TGCTGACAATCAGCCTCTTTT |
| ZF α-eHb_qF | 5'-TGCTCTCTCCAGGATGTTGA |
| ZF α-eHb_qR | 5'-TCACAGTCTTGCCGTGTTTC |
| ZF spi1_qF | 5'-GGGCAGTTTTAACCAAAGATCA |
| ZF spi1_qR | 5'-CCCAAGAGTGATCGTTCTGAC |
| ZF l-plastin exon9_qF | 5'-CGAAAACCAGGACATCGACT |
| ZF l-plastin exon9_qR | 5'-CCCCAGTGAGTTCATCCAGT |
| ZF rag1_qF | 5'-TCTCCAGACGATTCCGTTATGA |
| ZF rag1_qR | 5'-TGACCACCACAGTAAAGCCAGA |
| ZF rag2_qF | 5'-CAAACAGCTCTCAGATTTCG |
| ZF rag2_qR | 5'-CCAGGTCTAGTAAGGAGAAAC |
| ZF Lck_qF | 5'-AACAACCGTCAAGTGGCTATC |
| ZF Lck_qR | 5'-TGCGTGACCACAGCGAACAGT |
| ZF arhgef3_qF | 5'-ACTGCAGCGTGGACGAGT |
| ZF arhgef3_qR | 5'-CCCGAGACAGAGGCTTCAC |
| ZF emilin1a_qF | 5'-AGGGCAAGACCTACTGGTGA |
| ZF emilin1a_qR | 5'-CTCGAGTTGTCTAATCCTGTCG |
| ZF nbeal2_qF | 5' GTGGACTTTCCACCATCTCG |
| ZF nbeal2_qR | 5'-TGAGAGGGGCTGATACACG |
| ZF max_qF | 5'-TTGCAAGGGGAAAAGCAA |
| ZF max_qR | 5'-TTCTGCCTCTTCAGGTCGTC |
| ZF csf3r_qF | 5'-CAGCTGGAGTCTTTCGGAGA |
| ZF csf3r_qR | 5'-CTTAACTGCACAATGAAGGTCAA |
| ZF mpl_qF | 5'-ACCAACCTACCTTTAAGCAAAGAG |
| ZF mpl_qR | 5'-CTGGAGAAGAAAGAGATGACTGC |
| ZF epor_qF | 5'-TGTGGAGGACAGTCACATGG |
| ZF epor_qR | 5'-CTGTAAACCACGCAAAACCA |
| ZF tpo_qF | 5'-GCATCAGCATTCACAAAGCA |
| ZF tpo_qR | 5'-CCCAAAGACAGCAGGATCTC |
| ZF epo_qF | 5'-TGTTTTGCGAATGTTTCACG |
| ZF epo_qR | 5'-TCCACTCCAGCACCATCAG |
| ZF mpo_qF | 5'-GGGGCAGAAGAAGAAAGTCC |
| ZF mpo_qR | 5'-CCCTTGCTAAACTCTCATCTCG |
| ZF β-eHb_qF | 5'-AGGCTCTGGCAAGGTGTCTCA |
| ZF β-eHb_qR | 5'-CATTGGGTTTCCCAGGAT |
| ZF fli_qF | 5'-CAACGGATCCAGAGAGTCG |
| ZF fli_qR | 5'-CCATGTAGCCAGTATAGTTCATCTG |

**C. Supplementary Figure S1.** The map for pSYC-102-CALR vectors

**
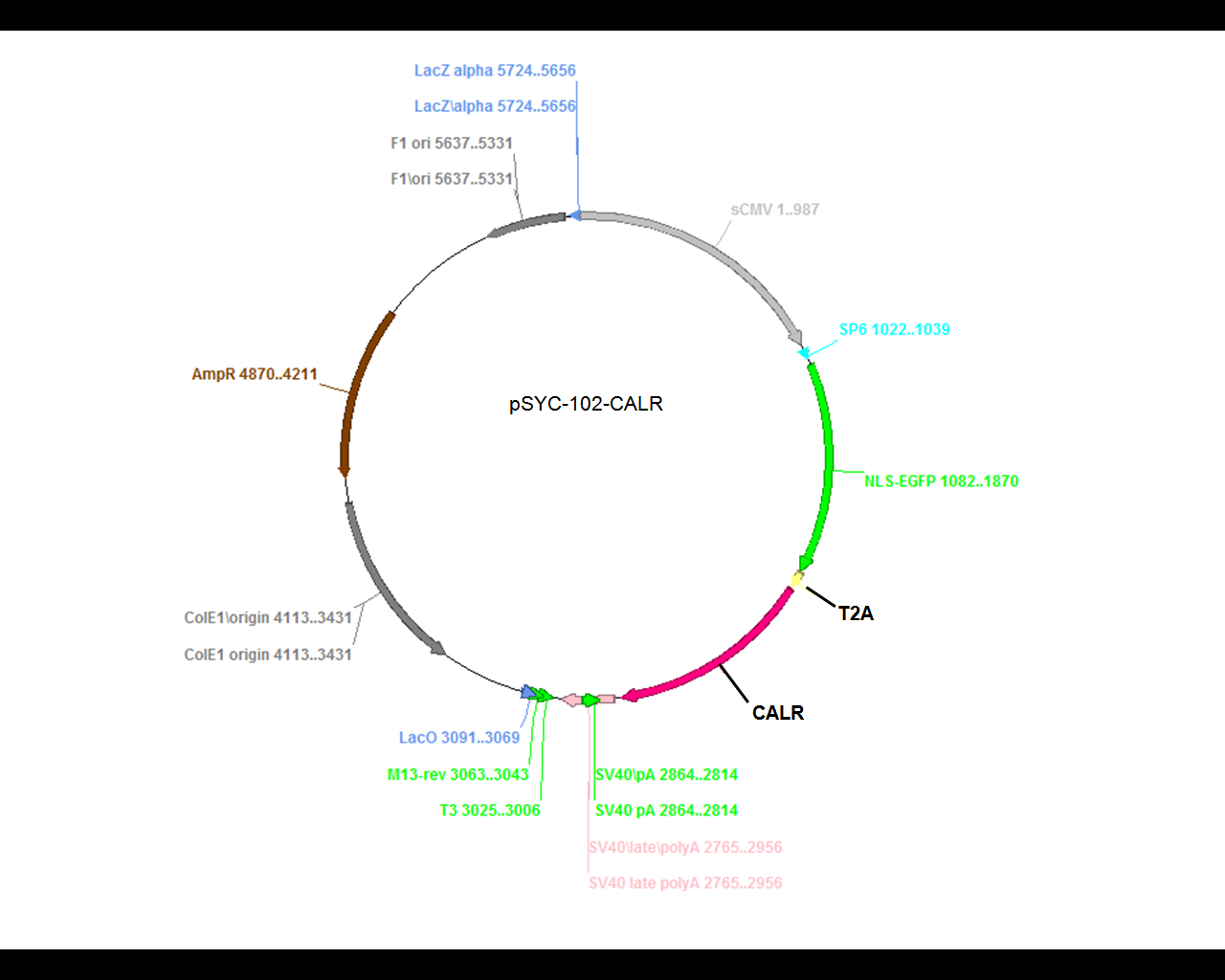
**
